# A Rare Case of Refractory Epilepsy Associated With Brain Calcifications and Mucocutaneous Candidiasis

**DOI:** 10.7759/cureus.83507

**Published:** 2025-05-05

**Authors:** Prashant Dubey, Prajwal Rao, Pravin Naphade

**Affiliations:** 1 Neurology, Rohilkhand Medical College, Bareilly, IND; 2 Neurology, Dr. D. Y. Patil Medical College, Hospital & Research Centre, Pune, IND

**Keywords:** addisons disease, autoimmune polygrandular syndrome type1, epilepsy, hypocalcemia, mucocutaneous candidiasis, seizure

## Abstract

Autoimmune polyendocrine syndrome type 1 (APS-1) is a rare and complex primary immunodeficiency disorder. The classic clinical triad of APS-1 includes chronic mucocutaneous candidiasis, hypoparathyroidism, and adrenal insufficiency. Clinically, APS-1 presents with significant variability and is characterized by autoimmune dysfunction affecting both endocrine organs (including the parathyroids, adrenal glands, thyroid, gonads, and pituitary) as well as non-endocrine tissues (such as the skin, liver, kidneys, lungs, eyes, and intestines).

Here we present a 23-year-old female with a history of abnormal body movement associated with posturing and transient loss of consciousness, along with a history of recurrent oral ulceration, itchy patches over intertriginous areas, and pigmentation of skin. Her examination was suggestive of low blood pressure, oral and cutaneous candidiasis, and hyperpigmentation of the skin. Routine investigations showed very low serum calcium, low parathyroid hormone (PTH) levels, and low early morning cortisol levels, and pathological calcification of the basal ganglia was noted on CT brain. As the patient met the diagnostic criteria of APS-1, she was treated accordingly and responded well.

## Introduction

Autoimmune polyendocrine syndrome type 1 (APS-1) is a rare and complex primary immunodeficiency disorder. The classic clinical triad of APS-1 includes chronic mucocutaneous candidiasis, hypoparathyroidism, and adrenal insufficiency [1,2]. While the overall prevalence of APS-1 is estimated at 10 cases per million people, certain populations, such as Iranian Jews and Finns, exhibit higher rates [3,4,5]. Clinically, APS-1 presents with significant variability and is characterized by autoimmune dysfunction affecting both endocrine organs (including the parathyroids, adrenal glands, thyroid, gonads, and pituitary) as well as non-endocrine tissues (such as the skin, hair, liver, kidneys, lungs, eyes, and intestines [6,7].

Autoimmune polyglandular syndrome type 1 arises from a failure in central immune tolerance, which leads to the development of autoimmunity. Since the identification of the *AIRE *gene, significant progress has been made in unraveling the underlying mechanisms of this condition. *AIRE *is predominantly expressed in medullary thymic epithelial cells and encodes a DNA-binding protein known as the autoimmune regulator. This protein plays a crucial role in promoting the expression of a wide range of tissue-specific antigens within the thymus. By exposing developing T cells to these antigens, the immune system is able to eliminate potentially self-reactive cells through negative selection. When *AIRE *is defective or absent, this screening process is impaired, allowing autoreactive T cells to bypass deletion and enter the peripheral circulation, where they can contribute to autoimmune disease [8].

## Case presentation

A 23-year-old female presented with a history of multiple episodes of seizures over the past three to four years. These seizures were characterized by abnormal body movements in which the patient had posturing of the upper limb, with spasmodic contraction of fingers, sometimes associated with the extension of the knee and ankle, involving all four limbs. There was also fainting, transient loss of consciousness lasting five to ten minutes, and occasional facial twitching. Additionally, she reported recurrent oral ulcers and difficulty swallowing food over the past five to six years.

There was no history of confusion, head-turning, or upward rolling of the eyes associated with the episodes of abnormal body movements. The patient did not report any episodes of tongue biting, urinary or fecal incontinence, or falls. Additionally, there were no premonitory symptoms or aura before the episodes. The frequency of these events was three to four episodes per day. There was no history of similar complaints in the family. The patient has a history of multiple hospital visits and consultations with various physicians. Upon presentation to our hospital, she was already on four antiepileptic medications (levetiracetam, lacosamide, clobazam, perampanel), yet she continued to experience episodes one to two times daily.

**Figure 1:**
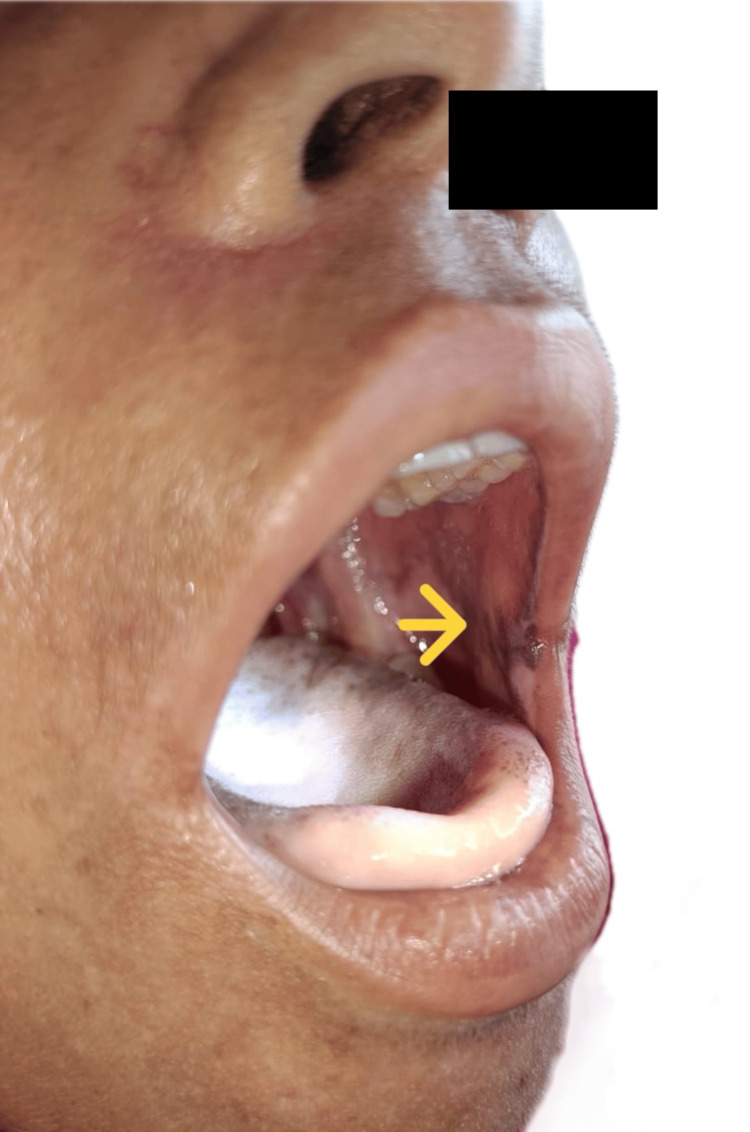
Hyperpigmentation inside the oral cavity. Yellow arrow pointing towards hyperpigmentation in oral cavity.

**Figure 2:**
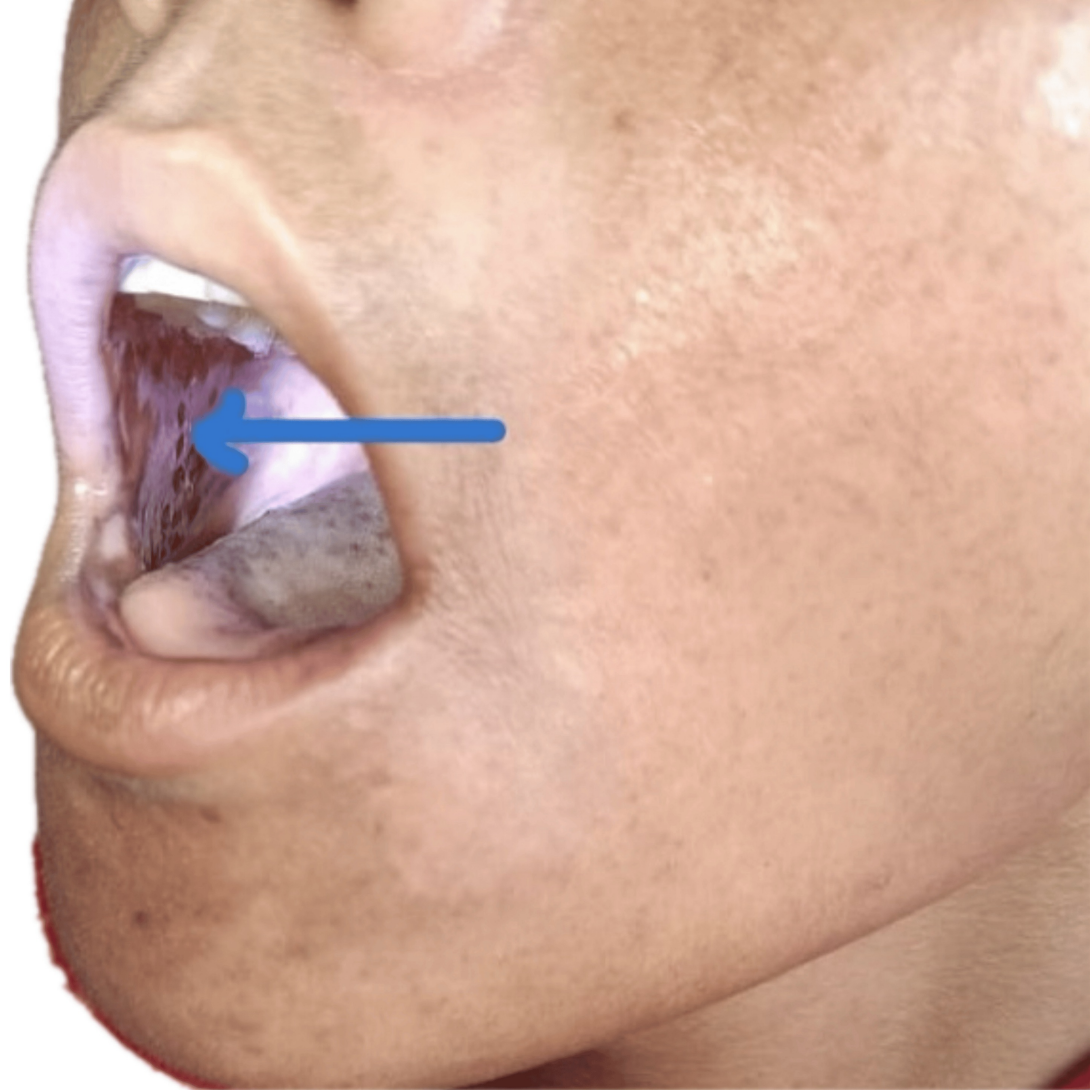
Whitish patches over buccal mucosa suggestive of oral candidiasis. Blue arrow pointing towards white patch in oral cavity.

**Figure 3:**
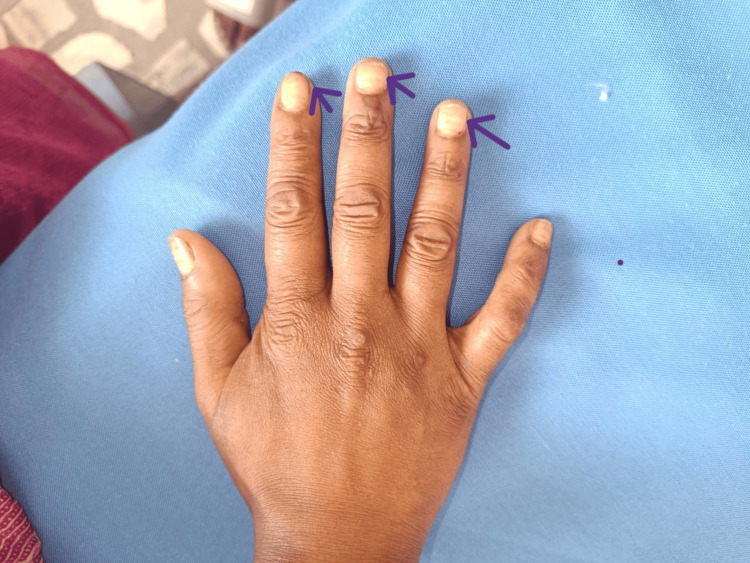
: Onycomycosis involving finger of right hand. Purple arrows pointing towards index, middle, ring  finger suggestive of onycomycosis.

**Figure 4:**
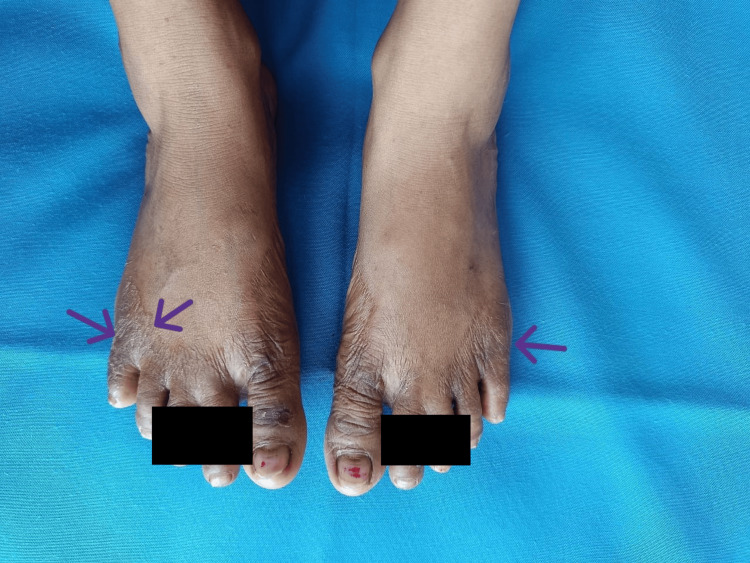
Cutaneous candidiasis involving lower limbs. Purple arrow pointing toward irregular patches over extensor aspect of both foot.

On examination, the patient presented with a blood pressure of 70/30 mmHg and a pulse rate of 126 beats per minute; however, the extremities were warm to the touch. No significant postural drop in blood pressure was noted. The general examination revealed hyperpigmentation of the skin over the malar region, elbows, and feet. In the oral cavity, multiple hyperpigmented patches were observed (Figure 1). Additionally, multiple whitish patches were noted in the oral cavity (Figure 2), axilla, inguinal area, nails (Figure 3), and the digits of the lower limbs (Figure 4), which were diagnosed as tinea cruris. An inducible carpal spasm was observed using a BP cuff, and a positive Chvostek sign was elicited on the face. Neurological examination, including assessment of higher mental functions, cranial nerves, motor and sensory systems, and reflexes, was normal.

Based on the history and examination, we evaluated the patient for recurrent carpal spasm and epilepsy. A baseline routine panel, including complete blood count, renal function tests, liver function tests, HbA1c, serum calcium, and ionized calcium, was ordered. Notable findings included a low serum calcium level of 4.3 mg/dL along with low ionized calcium0.6 mmol/l. Serum parathyroid hormone (PTH) level was also low. Given the patient's persistently low blood pressure and hyperpigmentation, an early morning serum cortisol level was measured, which was found to be low (Table 1).

**Table 1:** Investigation chart of the patient.

Test	Value	Reference value
Serum Calcium	4.3 mg/dl	(8.6-10) mg/dl
Ionic Calcium	0.6 mmol/l	(1.16-1.31)mmol/l
Parathyroid hormone	4 pg/ml	(15-65) pg/ml
Cortisol	2.9 mcg/dl	(4.82-19.5) mcg/dl
Anti TPO	0.88 IU/dl	<35 IU/dl
ANA by IFA	Negative	Normal
Follicular Stimulating Hormone	5.29 IU/L	(4.54-22.51) IU/L Mid cycle
LH	10.86 IU/L	(2.12-10.89) IU/L Mid cycle
Prolactin	8.98 ng/ml	(3.34-26.72) ng/ml
Serum testosterone	0.106 ng/ml	(0.06-0.86) ng/ml
Liver Function Test	Normal	
Kidney Function Test	Normal	
Complete Blood Count	Normal	
Total Urinary Calcium	11.25 mg/24hrs	100-300mg/24 hrs
Phosphorus	5.4 mg/dl	(2.5-4.5) mg/dl
Vitamin D3	28.37 ng/ml	Insufficient (10-30) Sufficient (30-100)ng/ml

**Figure 5:**
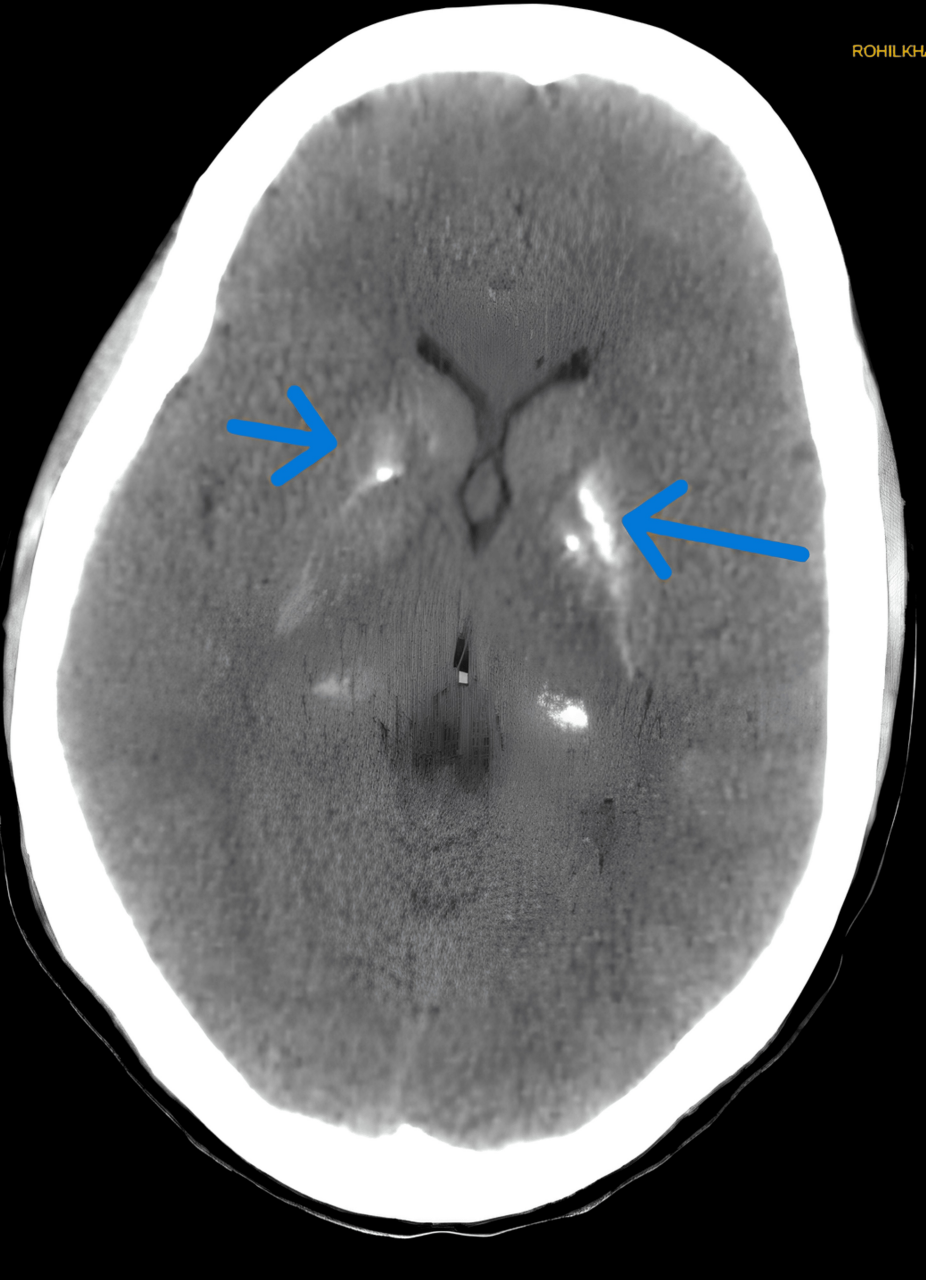
CT Brain showing hyperintense signal in bilateral basal ganglia and thalamus. Blue arrow pointing towards hyperintense signal in bilateral basal ganglia.

A cosyntropin stimulation test was performed, confirming persistently low cortisol levels, suggestive of primary adrenal insufficiency. CT brain was suggestive of bilateral basal ganglia calcification (Figure 5).

**Figure 6:**
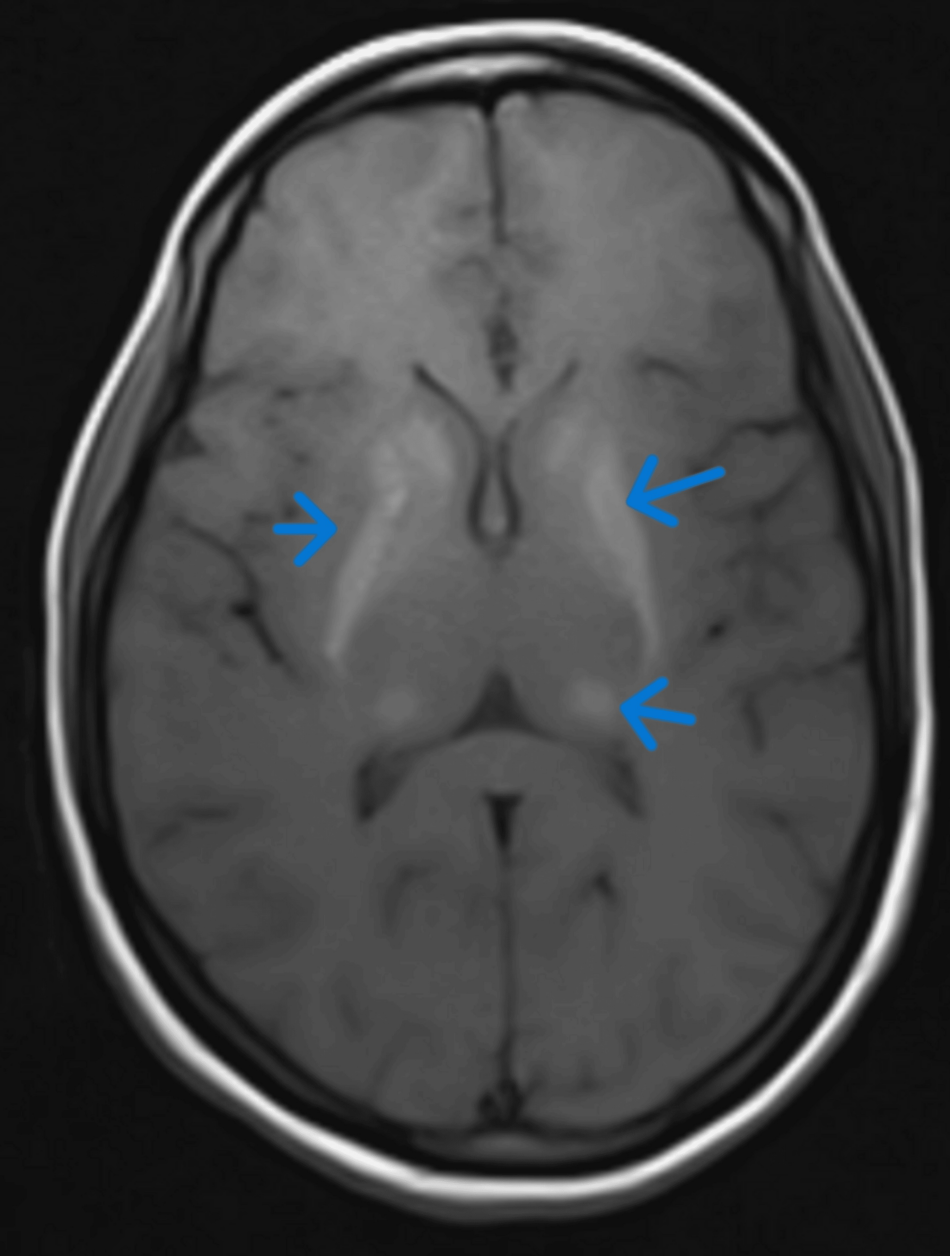
MRI brain T2 fluid-attenuated inversion recovery axial section showing hyperintensities in bilateral basal ganglia and thalamus. Blue arrow pointing toward hyperintense signal in basal ganglia and thalamus.

**Figure 7:**
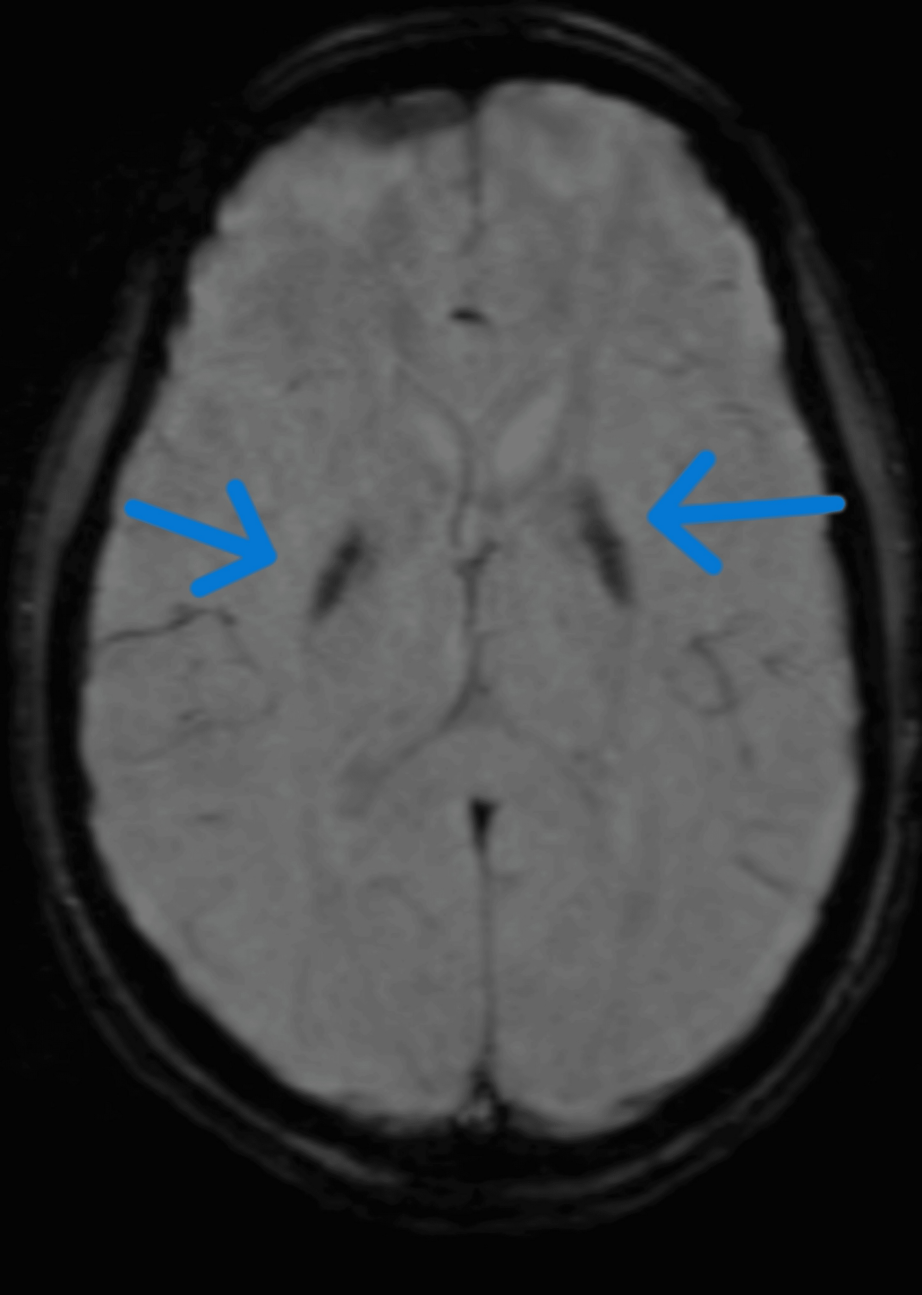
MRI brain susceptibility-weighted axial image showing blooming in bilateral basal ganglia. Blue arrow pointing toward bilateral basal ganglia.

MRI brain showed bilateral caudate, lentiform nuclei hyperintensity on T1/T2/fluid-attenuated inversion recovery (FLAIR) images, suggestive of pathological deposition of metabolite, which was likely calcium (Figures 6, 7).

Electroencephalogram (EEG)** **done at our centre was normal; the patient had no previous EEG. Differentials considered were Fahrs disease, hypoparathyroidism, and Addison's disease, but none of the above matched the complete clinical picture of the patient. A dermatology consult was obtained, which confirmed that the intertriginous skin lesions were consistent with cutaneous candidiasis, and the hyperpigmentation was indicative of Addison's disease. The patient, therefore, had all three components of the triad of autoimmune polyendocrine syndrome 1 (APS-1).

The patient was initiated on a regimen of calcium carbonate 1 g thrice a day, calcitriol 0.5 mg once a day, hydrocortisone 10 mg thrice a day, and fluconazole 100 mg once a day. Over the next 10 days, she showed gradual improvement, with complete resolution of seizures, carpal spasms, and syncopal episodes. She became normotensive. Consequently, all antiepileptic medications were discontinued. Genetic testing for *AIRE *gene mutations returned negative. Upon discharge, the patient was prescribed calcium carbonate, calcitriol, hydrocortisone, and fluconazole tablets. At her one-month follow-up, she reported no further episodes of carpal spasms or syncope. Additionally, her skin lesions were showing significant improvement, and she is progressing well towards recovery.

## Discussion

Autoimmune polyendocrine syndrome type 1 (APS-1) is an autoimmune disorder characterized by a triad of hypoparathyroidism, Addison's disease, and chronic mucocutaneous candidiasis. APS-1 is caused by mutations in the autoimmune regulator (*AIRE*) gene [8,9].

The most common initial manifestation of APS-1 is chronic mucocutaneous candidiasis, which typically presents in early childhood. Oral candidiasis may appear as redness and ulceration at the corners of the mouth, white or grey plaques, hyperkeratosis, or even tinea-like cutaneous lesions.

In APS-1, chronic mucocutaneous candidiasis (CMC) arises due to the presence of autoantibodies targeting key Th17 cytokines - IL-17A, IL-17F, and IL-22. These cytokines are essential for mucosal immunity, promoting the production of antimicrobial peptides and maintaining epithelial barrier integrity. The autoantibodies neutralize these cytokines, impairing the immune system's ability to control Candida infections on mucosal surfaces.

This phenomenon is unique to APS-1, as similar autoantibodies are not commonly found in other autoimmune conditions. The presence of these autoantibodies can be detected before the onset of CMC, suggesting a causal role in the development of the condition. This discovery has provided insight into the pathogenesis of CMC in APS-1 and underscores the importance of Th17 cytokines in mucosal immunity [10,11].

Hypoparathyroidism is the second most common feature of APS-1, typically presenting around the age of 5. It often appears earlier in females than in males [12]. Addison's disease generally develops later, typically a year or more after the onset of hypoparathyroidism, with symptoms including fatigue, abdominal pain, dizziness, weight loss, and hyperpigmentation. In females, a notable absence of axillary and pubic hair may also be observed [13]. Other possible manifestations of APS-1 include alopecia, keratitis, gastritis, pernicious anemia, autoimmune hepatitis, and autoimmune hypothyroidism [14].

The diagnosis of APS-1 is confirmed by identifying at least two of the three major components or by detecting mutations in the *AIRE *gene. The *AIRE *(autoimmune regulator) gene plays a vital role in establishing central immune tolerance by promoting the elimination of self-reactive T cells in the thymus. Mutations in this gene lead to a rare autosomal recessive disorder known as autoimmune polyendocrine syndrome type 1 (APS-1), characterized by multi-organ autoimmunity due to the failure to properly eliminate autoreactive immune cells. In approximately 8% of patients with APS-1, *AIRE *gene mutations may be negative. The *AIRE * gene plays a vital role in establishing central immune tolerance by promoting the elimination of self-reactive T cells in the thymus. Mutations in this gene lead to a rare autosomal recessive disorder known as autoimmune polyendocrine syndrome type 1 (APS-1), characterized by multi-organ autoimmunity due to the failure to properly eliminate autoreactive immune cells. Monoallelic mutations in the first plant homeodomain (PHD1) zinc finger of the *AIRE *gene have been identified in multiple families, exhibiting dominant inheritance patterns. These mutations are typically characterized by later onset, milder phenotypes, and reduced penetrance compared to classical APS-1. Unlike CARD or truncated AIRE mutants, which lack dominant-negative effects, these missense PHD1 mutations suppress gene expression driven by wild-type *AIRE *in a dominant-negative manner. These mutations are difficult to diagnose with normal genetic testing [15,16].

The treatment of APS-1 focuses on managing the various manifestations of the disease. Mucocutaneous candidiasis is treated with long-term antifungal therapy, typically for 4 to 6 weeks. Hypoparathyroidism is managed by addressing hypocalcemia through vitamin D supplementation, and recombinant parathyroid hormone (PTH) may be considered as an additional option. For adrenal insufficiency, the mainstay of treatment is glucocorticoid supplementation with hydrocortisone. Fludrocortisone can be used to replace mineralocorticoids as needed [17]. Overall, the current approach to managing APS-1 is symptomatic and supportive, addressing each specific manifestation of the disease [18].

In summary, our patient, initially treated for refractory epilepsy, was found to have hypocalcemia, which was the underlying cause of her seizures and carpopedal spasms. She presented with the full spectrum of APS-1, including mucocutaneous candidiasis, hypocalcemia-induced seizures, and adrenal insufficiency [19].

## Conclusions

APS-1 is a complex syndrome with multiple clinical manifestations. Patients presenting with seizures should be thoroughly evaluated for hypocalcaemia, and the underlying cause of hypocalcaemia should be investigated in detail. A comprehensive approach, including a detailed clinical history and examination, is crucial for diagnosing such rare disorders. Early diagnosis and prompt treatment are essential for reducing morbidity and improving the patient's quality of life.

## References

[1] Perheentupa J (2002 ). APS-I/APECED: the clinical disease and therapy. Endocrinol Metabol Clin North Am.

[2] Husebye ES, Perheentupa J, Rautemaa R, Kämpe O (2009). Clinical manifestations and management of patients with autoimmune polyendocrine syndrome type I. J Intern Med.

[3] Sato K, Nakajima K, Imamura H (2002). A novel missense mutation of AIRE gene in a patient with autoimmune polyendocrinopathy, candidiasis and ectodermal dystrophy (APECED), accompanied with progressive muscular atrophy: case report and review of the literature in Japan. Endocr J.

[4] Kisand K, Peterson P (2015). Autoimmune polyendocrinopathy candidiasis ectodermal dystrophy. J Clin Immunol.

[5] Meloni A, Furcas M, Cetani F (2008). Autoantibodies against type I interferons as an additional diagnostic criterion for autoimmune polyendocrine syndrome type I. J Clin Endocrinol Metab.

[6] Hedstrand H, Ekwall O, Haavik J (2000). Identification of tyrosine hydroxylase as an autoantigen in autoimmune polyendocrine syndrome type I. Biochem Biophys Res Commun.

[7] Ward L, Paquette J, Seidman E (1999). Severe autoimmune polyendocrinopathy-candidiasis-ectodermal dystrophy in an adolescent girl with a novel AIRE mutation: response to immunosuppressive therapy. J Clin Endocrinol Metab.

[8] Anderson MS, Venanzi ES, Klein L (2002). Projection of an immunological self shadow within the thymus by the AIRE protein. Science.

[9] Betterle C, Greggio NA, Volpato M (1998). Clinical review 93: autoimmune polyglandular syndrome type 1. J Clin Endocrinol Metab.

[10] Dittmar M, Kahaly GJ (2003). Polyglandular autoimmune syndromes: immunogenetics and long-term follow-up. J Clin Endocrinol Metab.

[11] Collins SM, Dominguez M, Ilmarinen T, Costigan C, Irvine AD (2006). Dermatological manifestations of autoimmune polyendocrinopathy-candidiasis-ectodermal dystrophy syndrome. Br J Dermatol.

[12] Sandru F, Petca RC, Dumitrascu MC, Petca A, Ionescu Miron AI, Baicoianu-Nitescu LC (2024). Cutaneous manifestations in autoimmune polyendocrinopathy-candidiasis-ectodermal dystrophy (APECED): a comprehensive review. Biomedicines.

[13] Gylling M, Kääriäinen E, Väisänen R (2003). The hypoparathyroidism of autoimmune polyendocrinopathy-candidiasis-ectodermal dystrophy protective effect of male sex. J Clin Endocrinol Metab.

[14] Ferré EM, Schmitt MM, Lionakis MS (2021). Autoimmune polyendocrinopathy-candidiasis-ectodermal dystrophy. Front Pediatr.

[15] Buzi F, Badolato R, Mazza C (2003). Autoimmune polyendocrinopathy-candidiasis-ectodermal dystrophy syndrome: time to review diagnostic criteria?. J Clin Endocrinol Metab.

[16] Ferre EM, Rose SR, Rosenzweig SD (2016). Redefined clinical features and diagnostic criteria in autoimmune polyendocrinopathy-candidiasis-ectodermal dystrophy. JCI Insight.

[17] Pellegrino M, Bellacchio E, Dhamo R, Frasca F, Betterle C, Fierabracci A (2018). A novel homozygous mutation of the AIRE gene in an APECED patient from Pakistan: case report and review of the literature. Front Immunol.

[18] Capalbo D, De Martino L, Giardino G (2012). Autoimmune polyendocrinopathy candidiasis ectodermal dystrophy: insights into genotype-phenotype correlation. Int J Endocrinol.

[19] Ahonen P, Myllärniemi S, Sipilä I, Perheentupa J (1990). Clinical variation of autoimmune polyendocrinopathy-candidiasis-ectodermal dystrophy (APECED) in a series of 68 patients. N Engl J Med.

